# The Potential of a Graphene Monolayer in Macrophage Polarization Using RAW 264.7 Cells

**DOI:** 10.3390/jfb17050232

**Published:** 2026-05-07

**Authors:** Iwona Lasocka, Karolina Gregorczyk-Zboroch, Aleksandra Krajewska, Ewa Skibniewska, Michał Skibniewski, Lidia Szulc-Dąbrowska

**Affiliations:** 1 Department of Biology of Animal Environment, Institute of Animal Science, Warsaw University of Life Sciences, 02-786 Warsaw, Poland; ewa_skibniewska@sggw.edu.pl; 2 Department of Preclinical Sciences, Institute of Veterinary Medicine, Warsaw University of Life Sciences, 02-786 Warsaw, Poland; karolina_gregorczyk-zboroch@sggw.edu.pl; 3 CENTERA, Centre for Advanced Materials and Technologies (CEZAMAT), Warsaw University of Technology, 02-822 Warsaw, Poland; aleksandra.krajewska@pw.edu.pl; 4 CENTERA Labs, Institute of High Pressure Physics PAS, 01-142 Warsaw, Poland; 5 Department of Morphological Sciences, Institute of Veterinary Medicine, Warsaw University of Life Sciences, 02-776 Warsaw, Poland; michal_skibniewski@sggw.edu.pl

**Keywords:** plasticity, cytocompatibility, wound healing

## Abstract

Maintaining an appropriate balance of macrophage subpopulations throughout the wound healing process, using a graphene monolayer as a substrate, may represent a promising therapeutic strategy. In this study, the effect of a graphene monolayer on the polarization of RAW 264.7 macrophages was investigated using flow cytometry, fluorescence microscopy, and ELISA. Analysis of surface M1 (MHC II, CD80, CD86) and M2 (CD163, CD200R, CD206) markers demonstrated generally higher expression of M1 markers in M1-polarized groups (control, CM1; and graphene monolayer, GM1) compared to M2-polarized groups (CM2 and GM2), likely as a result of LPS and IFN-γ stimulation. Culturing macrophages on a graphene monolayer as a substrate for LPS- and IFN-γ-stimulated cells was associated with a trend toward reduced expression of all analyzed M1-associated markers compared with the control M1 group; however, this effect did not reach statistical significance. TNF-α secretion was higher in GM1 compared to CM0, GM0, and CM2. In contrast, surface markers alone were less conclusive for identifying M2 polarization, whereas intracellular markers such as ARG1 provided a more robust indication of the M2 phenotype. ARG1 expression was significantly elevated in CM2 and GM2 groups, with GM2 showing a significant increase relative to the control groups (CM0, CM1) and GM0 and GM1. These findings further support ARG1 and NOS2 as reliable markers of M2 and M1 polarization, respectively. The graphene monolayer did not induce spontaneous macrophage polarization. Only under M1 (LPS and IFN-γ) and M2 (IL-4 and IL-13) stimulation did it show a consistent trend toward modest modulation of macrophage polarization, possibly creating conditions conducive to tissue healing.

## 1. Introduction

Macrophages are a type of white blood cell that represent a crucial first line of defense protecting the body from pathogens and tumor cells. Originating from circulating monocytes, macrophages migrate into tissues, where they differentiate into distinct, tissue-specific types, acquiring specialized functions shaped by their local environment [1]. The primary function of macrophages is phagocytosis, during which they respond to chemotactic signals and migrate to sites of infection, inflammation, or tissue injury, where they engulf pathogens and cellular debris and digest them intracellularly [2]. Moreover, they act as antigen-presenting cells (APCs) by displaying fragments of digested bacteria and other foreign substances on major histocompatibility complex (MHC) class II molecules on their cell surface to activate T cell-specific immune response [3]. Additionally, they secrete a wide range of signaling molecules, such as cytokines and chemokines, which regulate inflammation and recruit other immune cells to sites of infection or injury [4]. One of the key properties of macrophages is their capacity to dynamically adapt their phenotype in response to microenvironmental alterations, such as those occurring within a wound milieu, through polarization toward either the M1 (pro-inflammatory) or M2 (anti-inflammatory, reparative) phenotype. Classically activated (M1) macrophages arise in response to signals such as interferon (IFN)-γ and microbial products, e.g., lipopolysaccharide (LPS), which is a component of the cell wall of Gram-negative bacteria. Such stimulation leads to the activation of pro-inflammatory pathways that promote pathogen clearance and the initiation of inflammatory responses [5]. In contrast, alternatively activated (M2) macrophages are induced by signals associated with tissue repair and resolution of inflammation, supporting processes such as extracellular matrix remodeling, angiogenesis, and restoration of tissue homeostasis [6]. The balance and timely transition between these phenotypes are critical for proper wound healing and prevention of chronic inflammation [5]. In particular, IL-10, a key anti-inflammatory cytokine predominantly produced by M2 macrophages, plays a crucial role in regulating anti-inflammatory responses and in tissue repair and regeneration. Moreover, IL-10 suppresses the production of pro-inflammatory cytokines, thereby contributing to the resolution of inflammation [7]. Tumor necrosis factor-alpha (TNF-α), a pro-inflammatory cytokine, plays a dual role in wound healing [8]. On one hand, it contributes to pathogen clearance and provides essential signals that support the transition to the proliferative phase of healing. On the other hand, sustained exposure to TNF-α can be detrimental, as it promotes prolonged macrophage activation, leading to excessive production of inflammatory mediators. This persistent inflammatory state impairs macrophage polarization toward the M2 phenotype, which is crucial for tissue repair and reconstruction. The balance and timely transition between these phenotypes are critical for proper wound healing and prevention of chronic inflammation [5].

The introduction of nanomaterials with defined physicochemical characteristics as microenvironmental stimuli has the potential to modulate macrophage polarization. In this manner, control of macrophage polarization may become a strategy employed in wound-healing therapy. However, both the polarization process and wound healing itself are complex, and careful analysis of these processes is required. Maintaining an appropriate balance of macrophage subpopulations and their duration of action throughout the overlapping phases of wound healing (inflammation, proliferation, and remodeling) appears to be a critical factor. The second major research problem is finding a suitable biocompatible material that can initiate the macrophage polarization process and, consequently, accelerate wound healing. One noteworthy and promising proposal is the use of graphene. Its discovery in 2004 marked a new chapter in the applications of 2D carbon materials, and its properties have since been recognized for their antimicrobial activity and their capacity to promote wound healing, including when employed as a scaffold in wound dressings [9,10,11,12]. Scientific studies indicate that graphene and its derivatives may influence macrophage polarization [13,14,15]. However, the diversity of physical and chemical forms of graphene and its derivatives affects the obtained results, making it impossible to unequivocally determine the direction of polarization [16]. Nevertheless, certain patterns can be observed: depending on their size, graphene flakes can be internalized by macrophages and most often induce polarization toward the M1 phenotype [10,11], whereas functionalized graphene in the form of scaffolds directs polarization toward the M2 phenotype [9,12]. Cao et al. [13] reported that the nanotopographical characteristics of the TiO_2_ nanotubes loaded with graphene oxide (negatively charged and a large number of wrinkles) positively influence macrophage polarization toward the M2 phenotype, thereby promoting subsequent tissue regeneration. In contrast, Lin et al. [10] demonstrated that exposure of human M1 macrophages to few-layer graphene (FLG; 50 µg/mL) induced an increase in the production of pro-inflammatory cytokines and reactive oxygen species compared to the M1 control group.

The unequivocal identification of polarized macrophages remains unresolved; therefore, a combination of approaches is recommended, including the assessment of protein markers, cytokine/chemokine profiles, and gene expression [17]. In murine models, macrophage polarization can be characterized using both surface and intracellular markers. Classically activated (M1) macrophages are commonly identified by increased expression of MHC class II, CD68, CD80, and CD86, whereas alternatively activated (M2) macrophages are associated with CD163, CD200R, and CD206 expression. Intracellular markers further support this classification, with inducible nitric oxide synthase (iNOS/NOS2) as a hallmark of M1 polarization, and arginase 1 (Arg1), resistin-like molecule alpha (Fizz1), and chitinase-like protein 3 (YM1)—the latter two being murine-specific—indicative of the M2 phenotype. Functionally, M1 macrophages predominantly produce pro-inflammatory cytokines, including interleukin (IL)-1β, IL-6, (TNF)-α, and interferon (IFN)-γ, whereas IL-12 is not produced in RAW 264.7 cells in response to stimulation with lipopolysaccharide (LPS), a component of the cell wall of Gram-negative bacteria [18]. In contrast, M2 macrophages secrete anti-inflammatory mediators such as IL-10 and transforming growth factor (TGF)-β.

In this study, surface (MHC II, CD80, CD86, CD163, CD200R, and CD206) and intracellular (Arg1 and iNOS/NOS2) markers, as well as pro- (TNF-α) and anti-inflammatory (IL-10) cytokines, were assessed using flow cytometry, optical microscopy, and enzyme-linked immunosorbent assay (ELISA), an immunoenzymatic technique used to quantify protein levels.

Cell viability and morphology of cells cultured on a graphene monolayer substrate were also routinely evaluated. The studies cited above suggest that the graphene monolayer used in the present work may influence macrophage polarization or even directly induce it.

## 2. Materials and Methods

### 2.1. Graphene Monolayer

Graphene-coated glass slides were prepared using commercially available single-layer graphene on copper foil purchased from Graphenea (San Sebastián, Spain). The graphene was subsequently transferred from the copper substrate onto rounded glass cover slides by an electrochemical delamination process [19].

**Figure 1:**
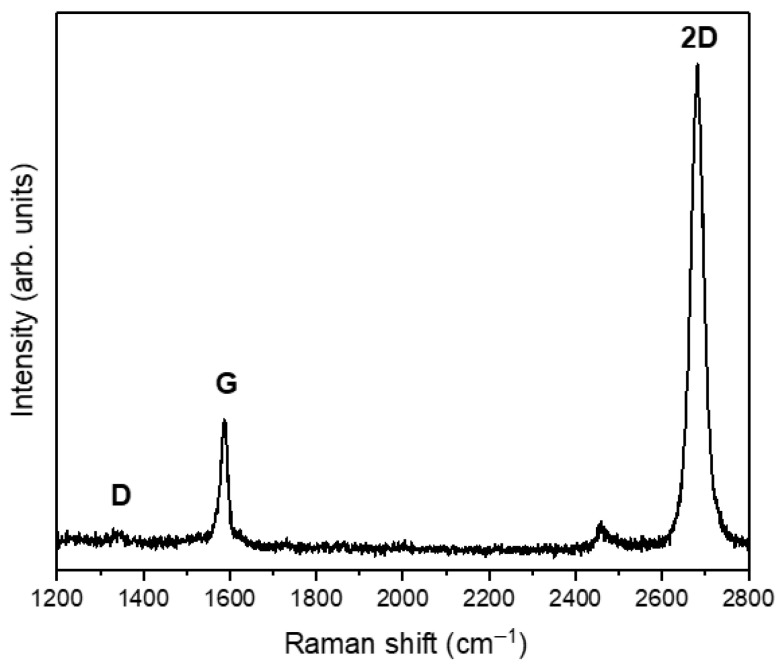
Raman spectrum of graphene transferred onto a glass slide.

One of the methods used to identify graphene monolayers is Raman spectroscopy, which was employed in the present study (Figure 1). Raman measurements were performed using a Renishaw InVia micro-Raman system (Renishaw, Wotton-under-Edge, UK) with a 532 nm laser as the excitation source.

### 2.2. Cell Cultures

RAW 264.7 macrophages (ATCC TIB-71) were obtained from the American Type Culture Collection (ATCC, Manassas, VA, USA). Cells were seeded on glass slide (control) and glass slide covered with graphene monolayer with cell density of 1 × 10^5^ cells/mL in RPMI supplemented with 10% fetal bovine serum (FBS, Sigma-Aldrich, Merck KGaA, Darmstadt, Germany), and 100 U/mL penicillin, and 100 μg/mL streptomycin (HyClone, Logan, UT, USA) in a 5% CO_2_ humidified atmosphere at 37 °C for 48 h.

**Figure 2:**
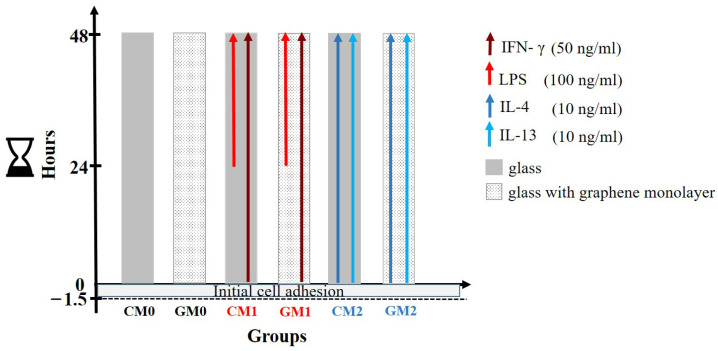
Schematic representation of macrophage polarization on control glass slides (C) and glass slides with a graphene monolayer (G). Following the initial adhesion phase (1.5 h), cells grown on both types of slides were left unstimulated (CM0 and GM0) or were stimulated with interferon-γ (IFN-γ) for 48 h, with lipopolysaccharide (LPS) introduced during the last 24 h to induce M1 po-larization (CM1 and GM1), or with interleukin-4 (IL-4) and IL-13 (IL-13) for 48 h to induce M2 polarization (CM2 and GM2).

LPS (100 ng/mL) and IFN-γ (50 ng/mL) were used to initiate macrophage polarization towards M1, while IL-4 (10 ng/mL) and IL-13 (10 ng/mL) (all from Sigma-Aldrich, Merck KGaA) were used to induce M2 polarization. M1 cells were stimulated with IFN-γ for 48 h, with LPS added during the final 24 h of the experiment. For M2 polarization, cells were stimulated with IL-4 and IL-13 (Figure 2). These two groups served as positive controls for M1 and M2 identification, respectively.

Before analyzing, macrophages were detached using 3 mM ethylenediaminetetraacetic acid (EDTA; Sigma-Aldrich, Merck KGaA) to avoid damage to surface markers. Control and positive control samples corresponding to macrophages cultured on a graphene monolayer were included in all assays (Figure 2).

### 2.3. Cell Size, Granularity, and Viability Assessment

The prepared cell suspension was analyzed using a BD LSRFortessa Cell Analyzer (Becton Dickinson and Company, San Jose, CA, USA), and the acquired data were analyzed using BD FACSDiva 7.0 software (Becton Dickinson and Company). RAW 264.7 size, granularity, and viability were determined using forward (FSC) and side (SSC) scatter and Zombie green viability dye (BioLegend, San Diego, CA, USA), respectively.

### 2.4. Wheat Germ Agglutinin (WGA) for Plasma Membrane Labeling

Cells were fixed with 4% paraformaldehyde (PFA; Sigma-Aldrich, Merck KGaA) for 15 min at 37 °C and then washed three times in PBS. WGA, Alexa Fluor Plus 568 conjugate (5.0 µg/mL) (Thermo Fisher Scientific, Waltham, MA, USA) was applied to cover cells adhering for 10 min at room temperature. The labeling solution was removed, and the cells were washed twice with PBS, then stained with Hoechst 33345 for DNA visualization. After washing in PBS, slides were closed with Glycergel Mounting Medium (Dako, Agilent Technologies, Santa Clara, CA, USA). Images were captured with an Olympus BX60 fluorescence microscope (Olympus, Tokyo, Japan) equipped with a PROMICAM 3-5CP camera and QuickPHOTO 2.3 software (Promicra, Prague, Czech Republic).

### 2.5. Flow Cytometry Analysis

The following surface antigens were selected to characterize M1 differentiation: CD80, CD86, MHC II, and M2 differentiation: CD163, CD206, and CD200R. Moreover, two intracellular antigens: NOS-2 (M1 phenotype) and Arg 1 (M2 phenotype) were analyzed following fixation and permeabilization using Cytofix/Cytoperm (BD Biosciences, San Jose, CA, USA).

The expression of surface and intracellular markers in RAW-264.7 macrophages was assessed by flow cytometry after 48 h exposure to a graphene monolayer. M1 markers (MHC II, CD80, and CD86) were analyzed as mean fluorescent intensity (MFI) using anti-mouse antibodies conjugated with PerCP, APC, and BV711, respectively. M2 markers (CD163, CD206, and CD200R) were evaluated using anti-mouse antibodies conjugated with PE, BV711, and APC, respectively. All antibodies were purchased from BD Biosciences.

### 2.6. Enzyme-Linked Immunosorbent Assay

The amount of TNF-α and IL-10 secreted by RAW 264.7 macrophages cultured on a graphene monolayer was quantified in the culture medium by enzyme-linked immunosorbent assay (ELISA) using OptEIA ELISA kits (BD Biosciences), according to the manufacturer’s instructions.

### 2.7. Statistical Analysis

All experiments were conducted in three independent replicates. The statistical analysis was performed using Statistica software (13.3 version) (TIBCO Statistica™, Statsoft, Poland). The mean values across the study groups were analyzed using Student’s *t*-test for the dependent variables. Differences between groups were analyzed at the *p* ≤ 0.05 significance level.

## 3. Results and Discussion

### 3.1. Graphene Monolayer Identification

The representative Raman spectrum of the graphene structure, shown in Figure 1, exhibits three characteristic bands: the G band at 1585 cm^−1^, the 2D band at 2680 cm^−1^, and a weak D band at 1350 cm^−1^. The relatively low Full Width at Half Maximum (FWHM) of the 2D band, approximately 32 cm^−1^, together with the markedly higher intensity of the 2D band compared with that of the G band (2D/G > 2), confirms the presence of monolayer graphene. In addition, the very low intensity of the D band indicates a low defect density, confirming the high structural quality of the graphene layer.

### 3.2. The Viability and Morphology of RAW 264.7 Macrophages Under Different Polarization Conditions on Graphene and Control Substrates

**Figure 3:**
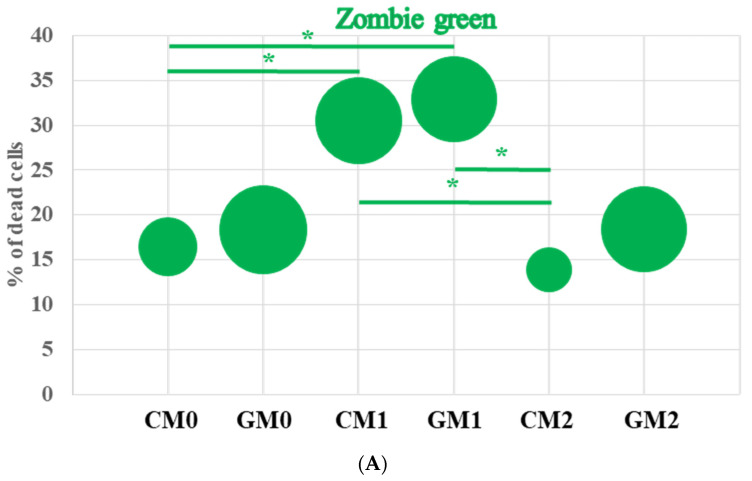

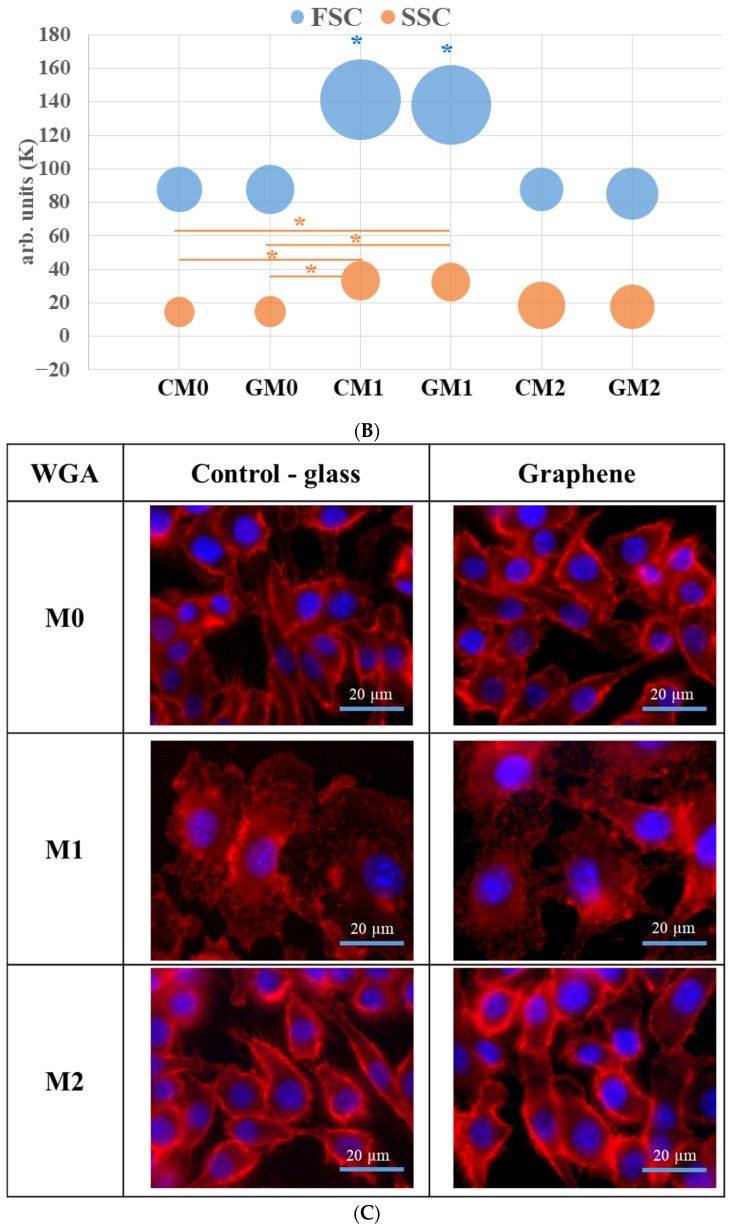
The viability and morphology of RAW 264.7 macrophages grown on glass slides (control) and glass slides coated with a graphene monolayer (graphene) after polarization into M1 and M2 phenotypes. (**A**) Viability was assessed using Zombie green staining and flow cytometry analysis. (**B**) Cell size and granularity were determined based on forward (FSC-A) and side (SSC-A) scatter profiles. The center of each circle represents the mean value, and the radius of the circle indicates the standard deviation within the group. (**C**) The morphology of RAW 264.7 macrophages was assessed using WGA labeling. Cells were stained with WGA (plasma membrane, red fluorescence) and Hoechst 33342 (DNA, blue fluorescence). An asterisk (*) above the circles indicates statistically significant differences compared with the other experimental groups.

Zombie Green dye was used to assess the effect of macrophage polarization and the presence of a graphene monolayer on cell viability. An increased level of macrophage death was observed in the CM1 and GM1 groups treated with LPS and IFN-γ (Figure 3A). 

Similar findings have been reported by other authors, who demonstrated increased macrophage death primarily as a consequence of LPS/IFN-γ stimulation [20]. The duration of our experiment was set at 48 h, as nanotoxicity studies are most commonly conducted within this timeframe [21]. Therefore, in the final experimental design, cells were stimulated with LPS only during the last 24 h of the experiment, together with IFN-γ, to induce M1 polarization. The LPS concentration applied in this study allows for comparative analyses not only in RAW macrophages, which are highly responsive to LPS stimulation, but also in other, less LPS-sensitive cell types, such as bone marrow–derived macrophages (BMDMs) [22]. The level of RAW 264.7 macrophage death in the CM0 and GM0 groups did not differ substantially; however, a higher standard deviation was observed in the GM0 group, which resulted in a lack of statistical significance when compared with the CM1 and GM1 groups. In turn, cell death in the CM2 and GM2 groups remained comparable to that observed in the control groups. Nevertheless, the GM2 group exhibited a higher standard deviation than CM2, a pattern that was also observed in the GM0 group. It may be speculated that the slightly increased variability and marginally higher level of macrophage death observed in the graphene groups could be associated with stronger cell adhesion to the graphene substrate, as previously suggested by other studies [23]. Enhanced adhesion may lead to more difficult cell detachment during sample preparation for flow cytometry, potentially resulting in mechanical damage to some cells. In contrast, Zhou et al. [24] reported a decrease in RAW 264.7 cell adhesion following exposure to conditioned medium collected from graphene-treated RAW 264.7 cells (20 μg/mL for 24 h). The authors suggested that negative feedback from the immune response induced by graphene-derived factors may play a critical role in preventing the overactivation of macrophages. Similarly, Huang et al. [25] observed that cell viability gradually decreased with increasing concentrations of graphene oxide quantum dots (GQDs) (from 5 to 80 μg/mL) over 24 h. It should be noted, however, that these two material forms—suspensions versus ground/scaffold—differ fundamentally in their properties and may therefore exert distinct effects on the cells under investigation. Indeed, it is well established that graphene and its derivatives in the form of dispersed flakes exhibit higher cytotoxicity at increasing concentrations compared to when they are incorporated into scaffolds [26].

Flow cytometric analysis of RAW 264.7 macrophage morphology revealed a significant increase in cell size (FSC-A) in the CM1 and GM1 groups compared with the remaining experimental groups (Figure 3B). The SSC-A parameter, which reflects cellular internal complexity or granularity, was also significantly increased (*p* ≤ 0.05) in CM1 and GM1 compared with the CM0 and GM0 groups. These observations indicate substantial changes in macrophage morphology, most likely resulting from LPS and IFN-γ stimulation. Interestingly, an increase in SSC-A values was also observed in macrophages polarized toward the M2 phenotype, although this change was not statistically significant. Importantly, culturing polarized macrophages on the graphene monolayer did not affect cell size or granularity compared with the corresponding polarized control groups (Figure 3B), suggesting that the graphene monolayer does not significantly influence RAW 264.7 macrophage morphology under the applied experimental conditions. Similar observations have been reported for other cell types cultured on graphene substrates, where graphene monolayers did not adversely affect cell viability [23] or membrane integrity [27].

WGA staining, which is commonly used for visualization of the plasma membrane, was applied to evaluate macrophage morphology and the continuity of the cell membrane [24]. In the present study, WGA labeling demonstrated a continuous and well-preserved plasma membrane contour in all analyzed experimental groups. However, in M1 macrophages (both control and graphene-cultured), an increased cell membrane ruffling was observed (Figure 3C). These morphological features are consistent with LPS-induced activation and cytoskeletal remodeling. Venter et al. [28] demonstrated that, in addition to actin cytoskeleton remodeling and the formation of filopodia and lamellipodia, LPS stimulation induces pronounced changes in cell spreading and membrane topography manifested as membrane ruffling.

In the broader context of biomolecule interactions with graphene monolayer, it will be particularly important in the future to apply vibrational spectroscopy methods, such as Raman and Fourier-transform infrared (FTIR) spectroscopy, to investigate biointerfacial interactions at the molecular level. As demonstrated in the study by Gerasimenko et al. [29], the combined use of spectroscopic approaches enables a deeper understanding of the mechanisms governing protein adsorption and cell–material interactions. In turn, the use of other imaging techniques, such as scanning electron microscopy (SEM) and transmission electron microscopy (TEM), will provide detailed insight into ultrastructural changes in intracellular organization and cell morphology of macrophages migrating on a graphene monolayer surface.

### 3.3. Effect of Polarization and Graphene Monolayer on the Release of Pro- and Anti-Inflammatory Cytokines by RAW-264.7 Macrophages

**Figure 4:**
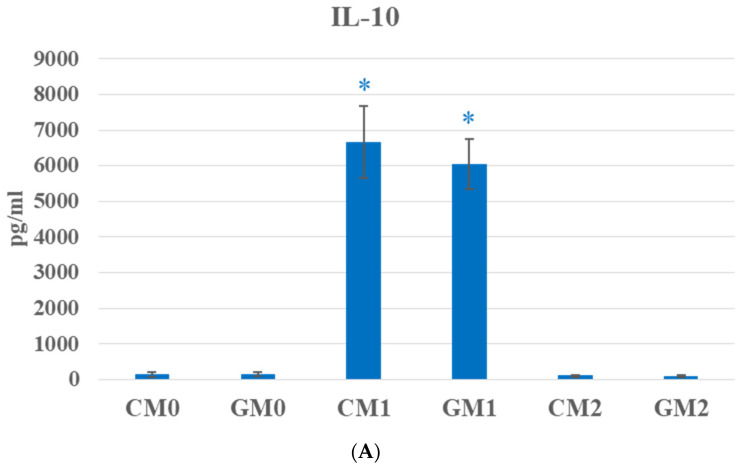

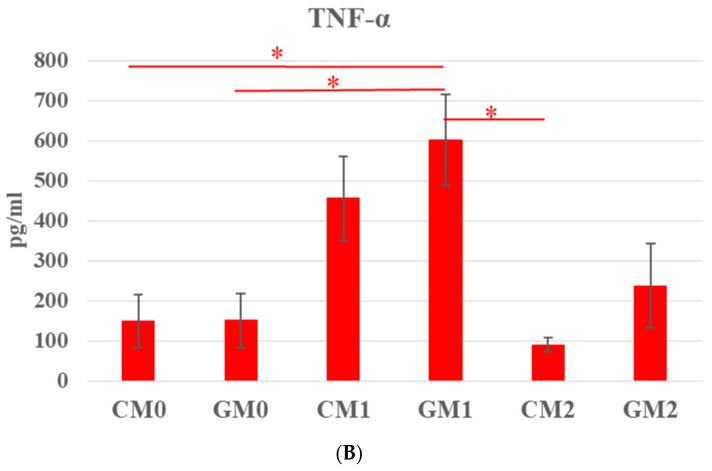
Cytokine secretion by RAW 264.7 growing on a graphene monolayer. IL-10 (**A**) and TNF-α (**B**) levels in cell supernatant after treatment with LPS and IFN-γ for M1 and IL-4 and IL-13 for M2 differentiation. * indicates statistically significant differences compared with the other experimental groups.

To evaluate the effect of graphene on macrophage polarization, two cytokines associated with macrophage functional phenotypes—IL-10 and TNF-α—were analyzed. In the present study, stimulation of macrophages with LPS resulted in a significant increase in IL-10 levels (Figure 4A). 

In contrast, no significant increase in IL-10 secretion was observed in the CM2 and GM2 groups compared with the respective control groups. Our observation is in line with previous reports demonstrating that RAW 264.7 macrophage-like cells often fail to significantly upregulate IL-10 under standard M2-polarizing conditions [30]. This can be attributed to the transformed nature of RAW 264.7 cells, which may exhibit altered cytokine production profiles compared to primary macrophages, including reduced or atypical IL-10 secretion upon polarization [30].

The kinetics of IL-10 and TNF-α secretion in RAW macrophages following LPS stimulation were previously described by Hobbs et al. [31]. In their study, macrophages stimulated with LPS (10 ng/mL) exhibited maximal TNF-α secretion at 48 h (11.7 × 10^3^ pg/mL), followed by a gradual decrease up to 72 h (7.88 × 10^3^ pg/mL). In contrast, IL-10 levels peaked earlier, at 24 h (621 pg/mL), and remained relatively stable at a slightly reduced level until 72 h (594 pg/mL). Importantly, the authors demonstrated that the early TNF-α response (observed within 6–16 h after stimulation) plays a critical role in determining the magnitude of the subsequent IL-10 response. However, the dynamics of this regulatory interaction may vary depending on the nature and strength of the inflammatory stimulus. In the present study, macrophages were stimulated not only with LPS at a substantially higher concentration but also with IFN-γ (50 ng/mL). Such combined stimulation is known to strongly promote M1 polarization and may alter cytokine secretion profiles. Therefore, the differences observed in the control groups without graphene, compared with those reported by Hobbs et al. [31], may be attributed to the higher LPS dose as well as the additional IFN-γ stimulation used in our experimental design.

GOQDs with an average size of approximately 7 nm were efficiently internalized by RAW 264.7 cells and attenuated TNF-α secretion following LPS stimulation (1 µg/mL) over a 24 h period [25]. In that study, TNF-α secretion in the LPS-stimulated control group reached approximately 1700 pg/mL [H], whereas in our experiments, it was 476 pg/mL in the CM1 group. It should be noted, however, that Huang et al. [25] employed a tenfold higher concentration of LPS than that used in our experiments. In our experimental setup, macrophages were stimulated with both LPS (100 ng/mL) and IFN-γ (50 ng/mL), and the incubation period was extended to 48 h. Under these conditions, no reduction in TNF-α secretion was observed in the presence of a graphene monolayer used as a substrate for macrophage culture (Figure 4B). Moreover, TNF-α secretion was significantly increased in the GM1 group compared with CM0, GM0, and CM2. Huang et al. [25] also reported elevated IL-10 secretion in the LPS-stimulated control group (>150 pg/mL) relative to the untreated control (~100 pg/mL), with GOQDs further enhancing cytokine production (~200 pg/mL). Interestingly, the increase reported in that study remained markedly lower than that observed in our CM1 and GM1 groups (Figure 4A). These discrepancies may stem from differences in the experimental design (24 h vs. 48 h), the additional IFN-γ stimulation applied in our study, as well as the distinct physical and chemical characteristics of the graphene materials used. Zhou et al. [24] revealed that graphene nanosheets (20 μg/mL for 24 h) enhance transcription and promote the secretion of pro-inflammatory factors in macrophages, even in the absence of additional polarization stimuli, including TNF-α. In experiments with RAW 264.7 cells, TNF-α secretion levels were markedly higher (8500 pg/mL) than in mouse (C57BL/6J) primary macrophages (~150 pg/mL), highlighting a fundamental difference in the responses of these two cell types to the nanoscale stimulus [24]. In contrast, Artiga et al. [32] reported that graphene nanoplatelets (10 or 50 μg/mL for 24 h) induced no significant changes in the levels of IL-6 (<100 pg/mL), IL-1β (<100 pg/mL), TNF-α (<200 pg/mL), IL-10 (<100 pg/mL), or IL-12 (<100 pg/mL) in human primary macrophages. These findings indicate that cytokine responses can vary not only between different forms of graphene but also between primary human and murine macrophages. This highlights a general challenge in studies of graphene-based nanomaterials and in comparing results, given the wide variety of graphene forms and variants, as well as differences in experimental design, exposure duration, and the sources of macrophages used.

### 3.4. Influence of Macrophage Polarization and Graphene Monolayer on Intracellular and Surface Marker Expression in RAW 264.7 Macrophages

**Figure 5:**
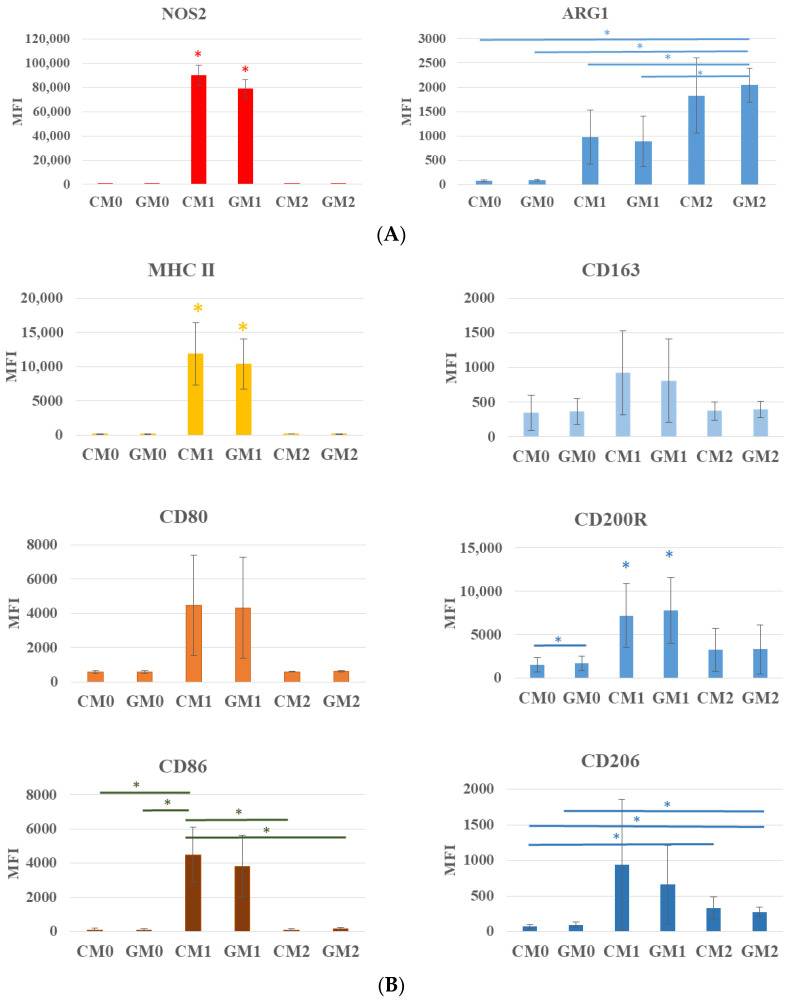
Expression of intracellular markers: NOS2 and ARG1 (**A**) and cell surface markers: MHCII, CD86, CD80 andCD163, CD200, CD206R (**B**) for M1 and M2 phenotype, respectively. MFI—mean fluorescence intensity. * indicates statistically significant differences compared with the other experimental groups.

To characterize M1 and M2 macrophage phenotypes, the expression of both intracellular (NOS2 and ARG1) and cell surface (MHC II, CD80, CD86, and CD163, CD200R, and CD206) markers was analyzed. The analysis of macrophage polarization revealed clear differences in marker expression between experimental groups. While surface marker analysis alone did not yield a definitive characterization of macrophage polarization, the expected M1 and M2 phenotypes were clearly confirmed through the evaluation of intracellular marker expression following stimulation (Figure 5A). 

Other studies have similarly underscored the importance of these markers as critical tools for identifying and confirming the direction of macrophage polarization [33,34].

In our study, NOS2 expression was significantly higher in the CM1 and GM1 groups compared with other experimental groups. Culturing macrophages on a graphene monolayer (GM1) resulted in a reduction in this parameter, although the decrease was not statistically significant. This trend was also observed for other analyzed surface markers, including MHC II, CD80, and CD86 (Figure 5B). For CD86 expression, a statistically significant difference was observed only in the CM1 group relative to the remaining groups, except for GM1.

While the differences in markers associated with M1 polarization were clearly visible in most cases for the CM1 and GM1 groups compared with the remaining groups, this pattern was less pronounced for markers characteristic of the M2 phenotype. Nevertheless, stimulation with LPS and IFN-γ resulted in an increase in the expression of CD163, CD206, and CD200R in both the CM1 and GM1 groups. The increased expression of selected M2-associated markers in M1-stimulated macrophages may reflect the high plasticity of macrophages and the activation of regulatory feedback mechanisms that limit excessive inflammatory responses, a phenomenon frequently observed in RAW 264.7 cells [35]. This effect was also reflected in the pattern of IL-10 secretion (Figure 4A).

Among the analyzed M2-associated markers, an increase was observed only for ARG1 expression in the CM2 and GM2 groups, with only the GM2 group showing a statistically significant difference compared with the remaining groups (CM0, GM0, CM1, and GM1) (Figure 5A). For surface markers (CD163, CD200R, CD206), expression was generally higher in the CM1 and GM1 groups compared with CM2 and GM2, particularly for CD200R, further supporting the phenomenon described above. In the case of CD206, a significant increase was detected in GM2 relative to CM0 and GM0, and between CM2 and CM0.

As demonstrated in the present in vitro study, RAW 264.7 macrophages do not fully recapitulate marker expression patterns observed in primary macrophages, such as BMDMs or monocyte-derived macrophages (MDMs). Tedesco et al. [36] also observed that canonical M2 markers, including CD206 and CD163, are weakly induced or inconsistently expressed in other widely used experimental models, such as THP-1-derived cells, and may not differ substantially between M1- and M2-polarizing conditions depending on the experimental setup. This highlights that marker expression in these models does not always follow the expected M1/M2 paradigm. Additionally, macrophage polarization is now recognized as a continuum rather than a strict binary classification. Considerable heterogeneity exists within M2 populations (e.g., M2a, M2b, M2c, M2d), with distinct and partially overlapping marker profiles. Therefore, reduced expression of selected canonical M2 markers does not necessarily indicate the absence of an M2-like phenotype but may instead reflect a shift toward a specific subtype or a mixed activation state under the applied experimental conditions.

Collectively, these results underscore that ARG1 is a reliable marker for monitoring M2 polarization, whereas NOS2 remains a robust indicator of M1 polarization. Notably, culturing macrophages on a graphene monolayer as a substrate for LPS- and IFN-γ-stimulated cells was associated with a trend toward reduced expression of all analyzed M1-associated markers compared with the control M1 group; however, this effect did not reach statistical significance. In contrast, the presence of a graphene monolayer significantly enhanced ARG1 expression following IL-4 and IL-13 stimulation, suggesting a potential substrate-mediated promotion of M2 polarization. Cao et al. [13] also demonstrated that, under LPS stimulation, the pro-inflammatory M1 response of murine macrophages (RAW 264.7) was attenuated, while M2 polarization was enhanced on TiO_2_ nanotubes functionalized with graphene oxide (GO). Specifically, they observed a significant decrease in the fluorescence intensity of iNOS and CD80, accompanied by a significant increase in CD163 and ARG, in the group treated with TiO_2_ nanotubes loaded with GO compared with the group not treated with graphene.

Graphene-based materials (G-BMs), particularly graphene oxide (GO), either alone or incorporated into composites, have been widely investigated for their potential to modulate macrophage polarization [16,37]. These materials have been shown to influence macrophage phenotype through both physicochemical surface properties and biointerfacial interactions [16,37,38], which may favor M2 polarization and promote wound healing. This highlights the importance of material design in regulating immune responses. However, this does not exclude the possibility that other materials within this group may exhibit similar properties. Our study contributes to this growing field by focusing on a graphene monolayer, providing additional insight into its influence on macrophage polarization.

In summary, the results indicate that the graphene monolayer did not induce spontaneous polarization of macrophages toward either the M1 or M2 phenotype. However, in the presence of stimulatory signals (LPS and IFN-γ or IL-4 and IL-13), it slightly changed the responses of both M1- and M2-polarized macrophages, possibly creating conditions conducive to tissue healing. Similar conclusions were reached by Hoyle et al. [39], who found that GO alone was not overtly pro-inflammatory in macrophages but inhibited IL-1β release from iBMDMs in a dose-dependent manner following LPS/ATP-induced inflammasome activation.

Further gene-level analyses are warranted to detect more subtle differences between M1 and M2 phenotypes and to elucidate the specific effects of the graphene monolayer. Moreover, assessing these differences across multiple time points would be essential for capturing temporal variations in the expression of genes characteristic of M1 and M2 phenotypes. As reported by Feito et al. [9], M1 and M2 macrophages (MØ) may coexist within the same microenvironment, and nanomaterials can modulate the M1/M2 balance in a time-dependent manner.

### 3.5. Potential Mechanism of Action of Graphene Monolayer on Polarized RAW 264.7 Macrophages

The surface interaction of a graphene monolayer with adherent macrophages is limited; however, as the surface area of LPS-stimulated macrophages increases, graphene interactions may also intensify. Consequently, a stronger anti-inflammatory effect can be expected, as graphene-based materials (G-BMs) have been shown to inhibit NF-κB activation in response to LPS [9,16,40,41]. Moreover, LPS itself may disrupt activation signals from TLR4 receptors. In contrast, under IL-4/IL-13 stimulation, which activates the STAT6 pathway, G-BMs have been shown to influence the expression of anti-inflammatory markers [9,13]. Additionally, the nanotopography of the graphene monolayer—such as wrinkles, ripples, and crumples—can affect cytoskeletal reorganization and surface receptor signaling, favoring an M2-like phenotype [16].

## 4. Conclusions

Based primarily on intracellular markers NOS2 and Arg1, it can be concluded that only under M1 (LPS and IFN-γ) and M2 (IL-4 and IL-13) stimulation, the graphene monolayer showed a consistent trend toward modest modulation of RAW 264.7 polarization. The obtained results suggest that graphene monolayers may support a shift toward an anti-inflammatory, tissue-repair-associated M2 phenotype, indicating their potential relevance for wound-healing applications. However, these changes did not reach statistical significance and should therefore be interpreted with caution. Therefore, further studies, including those using primary cells and additional macrophage models, are required to validate these findings.

## Data Availability

All detailed data are available upon request from the corresponding authors.

## Abbreviations

The following abbreviations are used in this manuscript:

MHC II	Major Histocompatibility Complex class II
CD80	Cluster of differentiation 80
CD86	Cluster of differentiation 86
CD163	Cluster of differentiation 163
CD200R	Cluster of differentiation 200 Receptor
CD206	Cluster of differentiation 206
ARG1	Arginase 1
NOS2/iNOS	Nitric Oxide Synthase 2
LPS	Lipopolysaccharide
IFN-γ	Interferon-Gamma
IL-4	Interleukin-4
IL-13	Interleukin-13
FLG	Few-Layer Graphene
CD68	Cluster of Differentiation 68
Fizz1	Resistin-Like Molecule Alpha
YM1	Chitinase-like Protein
IL-1β	Interleukin-1 Beta
IL-6	Interleukin-6
TNF-α	Tumor Necrosis Factor-alpha
IL-10	Interleukin-10
IL-12	Interleukin-12
WGA	Wheat Germ Agglutinin
APC	Allophycocyanin
PE	Phycoerythrin
BV711	Brilliant Violet 711
PerpCP	Peridinin-Chlorophyll-Protein
BMDMs	Bone Marrow-Derived Macrophages
GQODs	Graphene Oxide Quantum Dots
GO	Graphene Oxide
iBMDMs	Immortalized Bone-Marrow-Derived Macrophages
FSC-A	Forward Scatter Area
SSC-A	Side Scatter Area
G-BMs	Graphene-Based Materials
